# Trends in horizontal gene transfer research in *Salmonella* antimicrobial resistance: a bibliometric analysis

**DOI:** 10.3389/fmicb.2024.1439664

**Published:** 2024-09-12

**Authors:** Jin Yan, Benoît Doublet, Agnès Wiedemann

**Affiliations:** ^1^Department of Gastroenterology, The Second Xiangya Hospital of Central South University, Changsha, China; ^2^Research Center of Digestive Disease, Central South University, Changsha, China; ^3^IRSD - Institut de Recherche en Santé Digestive, Université́ de Toulouse, INSERM, INRAE, ENVT, UPS, Toulouse, France; ^4^ISP, INRAE, Université de Tours, Nouzilly, France

**Keywords:** *Salmonella*, antimicrobial resistance, horizontal gene transfer, CiteSpace, VOSviewer

## Abstract

Horizontal gene transfer (HGT) favors the acquisition and spread of antimicrobial resistance (AMR) genes in *Salmonella*, making it a major public health concern. We performed a bibliometric analysis to provide the current landscape of HGT in research on *Salmonella* AMR and identify emerging trends and potential research directions for the future. Data were collected from the Web of Science Core Collection and limited to articles and reviews published between 1999 and 2024 in English. VOSviewer 1.6.19 and CiteSpace 6.2.R1 software were used to conduct bibliometric analysis and visualize co-occurring keywords. A total of 1,467 publications were retrieved for analysis. American researchers contributed the most articles (*n* = 310). In the meantime, Institut National de Recherche pour l’Agriculture, l’Alimentation et l’Environnement have the highest citation/publication rate of 85.6. Recent studies have focused on the application of whole genome sequencing (WGS), *Salmonella* quinolone and colistin resistance, and the biocontrol of *Salmonella* AMR. These findings provide new insights into the role of HGT and help identify new targets for controlling the spread of AMR in *Salmonella* populations.

## Introduction

The discovery and use of antibiotics have promoted the prevention and treatment of bacterial infections but have simultaneously resulted in increased antimicrobial resistance (AMR). It is estimated that by 2050, deaths caused by AMR will increase to 10 million per year, resulting in a cost of 100 trillion USD (Despotovic et al., 2023). AMR can arise from mutations in chromosomal DNA or acquisition of AMR genes. Horizontal gene transfer (HGT) is one of the most important mechanisms for the acquisition and spread of AMR genes among bacteria. Among the different HGT mechanisms, conjugation is considered the main driver of resistance gene exchange between bacteria. The majority of medically relevant AMR genes are clustered on mobile genetic elements such as gene cassettes, transposons, genomic islands, and plasmids. Consequently, such resistance genes are easily swapped between bacteria in the same habitat, such as *Enterobacteriaceae* in the intestine of animals and humans (Tao et al., 2022).

*Salmonella enterica* serovars are prevalent human and animal pathogens that are responsible for gastroenteritis and typhoid diseases. It is responsible for 550 million cases of diarrhea annually, of which 220 million are under the age of five. Many of these cases are life threatening and deadly. Multi-drug resistance (MDR) *Salmonella* has been classified according to the World Health Organization as a pathogen of high priority, linked to the emergence of fluoroquinolone resistance (WHO publishes list of bacteria for which new antibiotics are urgently needed, 2017). In the context of HGT, *Salmonella* participates either as a donor or recipient of resistance genes, and is therefore implicated in the spread of resistance genes. It has been identified as a major driver of the rapid dissemination of AMR in both humans (Winokur et al., 2001) and animals (Mathew et al., 2009).

Bibliometric analysis is a quantitative research method that involves analysis and evaluation of scientific publications using statistical methods (Ellegaard and Wallin, 2015). This allows for the identification of important authors, institutions, and research trends in the field. By analyzing publication patterns and citation networks, researchers can identify important research gaps, emerging topics, and influential studies. Bibliometric analysis has been applied to the field of AMR (Sun et al., 2022). However, no bibliometric analysis has been conducted on HGT in *Salmonella* AMR. Therefore, it is necessary to provide a quantitative framework for evaluating the growth and impact of research on *Salmonella* AMR and HGT.

In this study, a bibliometric analysis was conducted in the area of *Salmonella* AMR to provide the current landscape of HGT research and to identify emerging trends and potential future research directions.

## Materials and methods

### Data collection and retrieval strategies

Data were collected from the Web of Science Core Collection (WoSCC) on May 27, 2024. The search query used was ((TS = (“horizontal gene transfer”) OR TS = (HGT) OR TS = (conjugation) OR TS = (transduction) OR TS = “mobile genetic element”) OR TS = (“mobile element”) OR TS = (“conjugative plasmid”) OR TS = (“mobilizable plasmid”) OR TS = (“integrative conjugative element”) OR TS = (ICE) OR TS = (“integrative mobilizable element”) OR TS = (IME) OR TS = (“transposable element”) OR TS = (transposon) OR TS = (phage) OR TS = (prophage) OR TS = (bacteriophage)) AND TS = (*Salmonella*) AND (TS = (“antibiotic resistance”) OR TS = (“antimicrobial resistance”) OR TS = (AMR) OR TS = (“acquired resistance gene”) OR TS = (“drug resistance”) OR TS = (“multidrug resistance”)). The results were limited to articles and reviews published in English and indexed in the Science Citation Index Expanded database.

### Data analysis

All acquired articles underwent a meticulous screening process to assess their relevance to HGT in *Salmonella* AMR. Variables such as the publication date, document type, affiliation, country, title, and keywords were extracted. Trend analysis was performed to discern patterns in scientific output across the temporal axes, citation indices, and author/institution/country contributions. Bibliometric analyses and network visualizations were performed using VOSviewer version 1.6.19 and CiteSpace version 6.2.R1 (64-bit) to construct networks of co-occurring keywords.

## Results

### Publication characteristics

**Figure 1:**
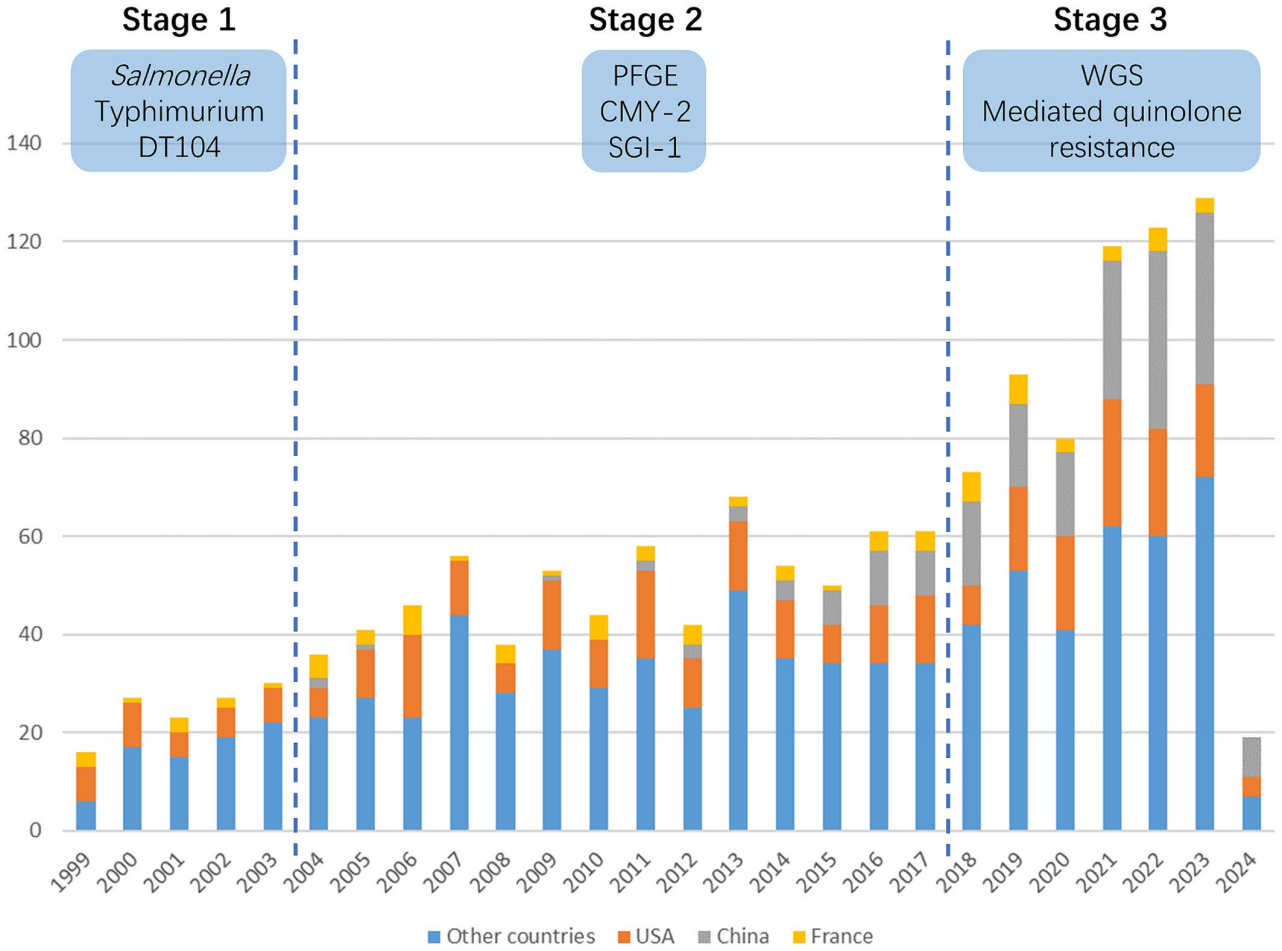
Trends in publications on horizontal gene transfer in *Salmonella* antimicrobial resistance. This figure displays the annual count of publications related to horizontal gene transfer in *Salmonella* antimicrobial resistance based on a dataset comprising 1,467 publications gathered from the WoS core collection spanning from 1999 to May 27, 2024 (refer to Materials and Methods for search query details). The figure delineates three distinct stages characterized by varying publication volumes, along with their corresponding research focal points. Additionally, contributions from the USA, China, and France were visually differentiated through distinct color highlights.

The WoSCC database contains a total of 1,467 publications related to AMR/HGT research in *Salmonella* from 1999 to 2024. The number of publications in this field has shown an increasing trend over the years (Figure 1). They were written by 7,028 authors from 1887 organizations in 105 countries. When considering the aging of references in this field, the Price’s index (de Price, 1969) and citing half-life were calculated.


$$Price^{\prime }s\;index=\frac{number\;of\;citations\;less\;than\;5\;years\;old}{total\;number\;of\;citations}\times 100\%$$

The Price’s index of these cited references is 17.5%, which means that 17.5% of them were from the last 5 years. The cited half-life is the median age of articles cited in the Journal Citation Report (JCR) year (Garfield, 2001). This indicates the turnover rate of the body of work on a subject. The citing half-life was 8 years. These two indices indicate that the aging of publications in this area is relatively slow, and research is stable and mature. The 1,467 publications received 52,591 citations, and the citation/publication in this area was 35.85. The h-index of retrieved publications was 102.

### Authors, countries/regions and institutions cooperation analysis

Table 1 shows the top 10 high-productivity authors and provides an overview of the most productive and cited authors in the field. The top 10 countries that made the most significant contributions to the field of HGT in *Salmonella* AMR are listed in Table 2. The USA scholars have contributed the most research papers in this field (310 papers in total). China follows with 201 publications, but the average citation/publication rate is only 20.8. France has the highest average citation/publication rate (63.8). The institutional analysis corroborates this finding. The distribution of outstanding institutions in this area is relatively equal among countries (Table 3). Three of the institutions were from the USA and contributed 50% of the total publications. The U.S. Food and Drug Administration (US FDA) published the most papers, with a citation/publication of 41.9. The Institut National de Recherche pour l’Agriculture, l’Alimentation et l’Environnement (INRAE) in France contributed only 27 publications but received 2,311 citations, with the highest citation/publication rate of 85.6.

**Table 1:** Top 10 high-productive authors on horizontal gene transfer in *Salmonella* antimicrobial resistance.

Rank	Author	Country	Publication	Citation	Citation/Publication	H index
1	Xu, Xuebin	China	19	210	11.1	21
2	Zhao, Shaohua	USA	18	618	34.3	37
3	Fanning, Seamus	Ireland	18	398	22.1	51
4	Foley, Steven L.	USA	17	438	25.8	24
5	Frye, Jonathan G.	USA	16	525	32.8	26
6	Hendriksen, Rene S.	Denmark	15	492	32.8	42
7	Cloeckaert, Axel	France	15	1,009	67.3	36
8	Guerra, Beatriz	German	14	610	43.6	36
9	Dougan, Gordon	England	14	1,245	88.9	82
10	Weill, Francois-Xavier	France	14	720	51.4	48

**Table 2:** Top 10 high-productive countries on horizontal gene transfer in *Salmonella* antimicrobial resistance.

Rank	Country	Publication	Citation	Citation/Publication
1	USA	310	13,935	45.0
2	China	201	4,184	20.8
3	England	147	6,666	45.3
4	Spain	96	4,019	41.9
5	France	82	5,232	63.8
6	Germany	80	4,587	57.3
7	Canada	78	3,678	47.2
8	South Korea	72	1,178	16.4
9	Italy	69	3,375	48.9
10	Denmark	61	2,769	45.4

**Table 3:** Top 10 most productive institutions on horizontal gene transfer in *Salmonella* antimicrobial resistance.

Rank	Institution	Country	Publication	Citation	Citations/Publication
1	U.S. Food and Drug Administration (US FDA)	USA	73	3,061	41.9
2	U.S. Department of Agriculture Agricultural Research Service (USDA ARS)	USA	50	1,710	34.2
3	University Oviedo	Spain	35	1,083	30.9
4	Center for Disease Control and Prevention	USA	29	1,723	59.4
5	Technical University of Denmark	Denmark	29	1,728	59.6
6	Pasteur Institute	France	29	1,364	47.0
7	Institut National de Recherche pour l’Agriculture, l’Alimentation et l’Environnement (INRAE)	France	27	2,311	85.6
8	South China Agricultural University	China	26	376	14.5
9	Yangzhou University	China	26	164	6.3
10	German Federal Institute for Risk Assessment	Germany	24	1,033	43.0

### Highly-cited papers analysis

Table 4 shows the top 10 most-cited papers related to this topic. Most were research articles with only one review. The most cited paper is “Epidemic multiple drug resistant *Salmonella* Typhimurium causing invasive disease in sub-Saharan Africa have a distinct genotype” (Kingsley et al., 2009) which has 391 citations. Most of them were published in journals specifically in the microbiology or antimicrobial fields.

**Table 4:** Top 10 highly-cited papers on horizontal gene transfer in *Salmonella* antimicrobial resistance.

Rank	Title	Citations	Year	First author	Type	Journal	JCR	IF
1	Epidemic multiple drug resistant *Salmonella* Typhimurium causing invasive disease in sub-Saharan Africa have a distinct genotype	391	2009	Robert A Kingsley	Research Article	Genome Research	Q1	7
2	Characterization of multiple-antimicrobial-resistant *Salmonella* serovars isolated from retail meats	342	2004	Sheng Chen	Research Article	Applied And Environmental Microbiology	Q2	4.4
3	Complete nucleotide sequence of a 43-kilobase genomic island associated with the multidrug resistance region of *Salmonella enterica* serovar Typhimurium DT104 and its identification in phage type DT120 and serovar Agona	325	2001	David Boyd	Research Article	Journal of Bacteriology	Q3	3.2
4	Molecular characterization of an antibiotic resistance gene cluster of *Salmonella* Typhimurium DT104	272	1999	Connie E. Briggs	Research Article	Antimicrobial Agents and Chemotherapy	Q2	4.9
5	Animal and human multidrug-resistant, cephalosporin-resistant *Salmonella* isolates expressing a plasmid-mediated CMY-2 AmpC beta-lactamase	233	2000	P. L. Winokur	Research Article	Antimicrobial Agents and Chemotherapy	Q2	4.9
6	Epidemic *Salmonella* Typhimurium DT 104--a truly international multiresistant clone	230	2000	E. John Threlfall	Review	Journal of Antimicrobial Chemotherapy	Q2	5.2
7	Antimicrobial resistance in non-typhoid *Salmonella* serotypes: a global challenge	202	2004	Lin-Hui Su	Review	Clinical Infectious Diseases	Q1	11.8
8	Emergence of domestically acquired ceftriaxone-resistant *Salmonella* infections associated with AmpC beta-lactamase	198	2000	Eileen F. Dunne	Research Article	Jama-journal of The American Medical Association	Q1	120.7
9	The *Salmonella* genomic island 1 is an integrative mobilizable element	196	2005	Benoît Doublet	Research Article	Molecular Microbiology	Q2	3.6
10	Plasmid-mediated quinolone resistance in non-Typhi serotypes of *Salmonella enterica*	194	2006	Kathryn Gay	Research Article	Clinical Infectious Diseases	Q1	11.8

JCR, Journal Citation Report; IF, Impact factor.

### Keyword clustering, burst, and evolution analysis

**Figure 2:**
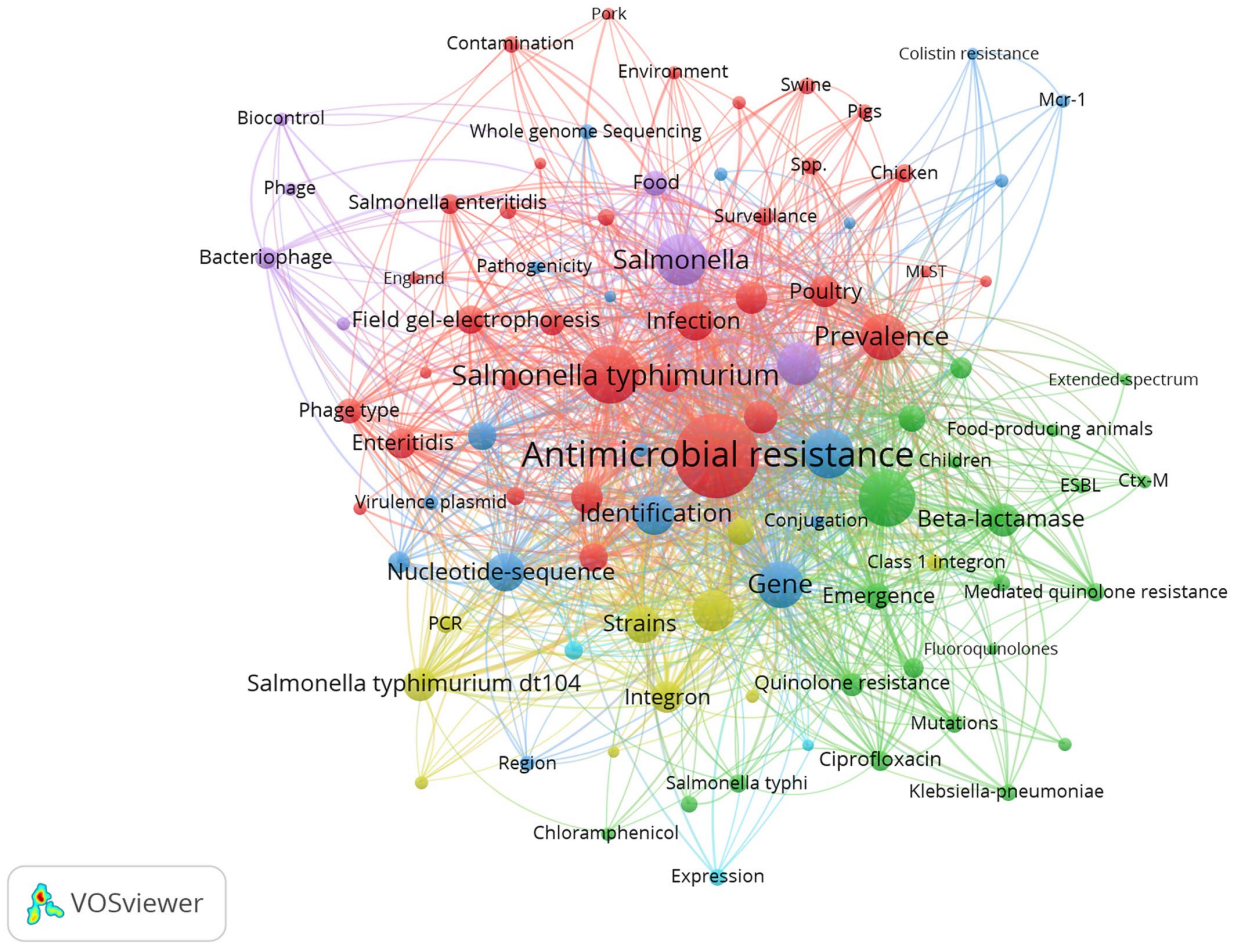
The co-occurrence of keywords. The size of each circle corresponds to the number of publications, with larger circles indicating higher publication counts. Connecting lines signify co-occurrence between keywords, with thicker lines signifying more frequent co-occurrences.

In Figure 2, the five clusters comprehensively map the intricacies of AMR in *Salmonella*, thereby emphasizing the role of HGT. The “Red” cluster deals with the epidemiological spread, including factors from various hosts to environmental transmission. The “Green” cluster focus on specific AMR mechanisms and their impact on clinical treatments. The “Blue” group dissects the molecular genetics that facilitate resistance, applying high-level sequencing techniques to decode these processes. The “Yellow” cluster is oriented around methodological tools and particular genetic elements like integrons that enable the study of resistance mechanisms. The “Purple” group delves into biocontrol strategies involving bacteriophages and the role of genomics in understanding *Salmonella enterica* in food systems.

**Figure 3:**
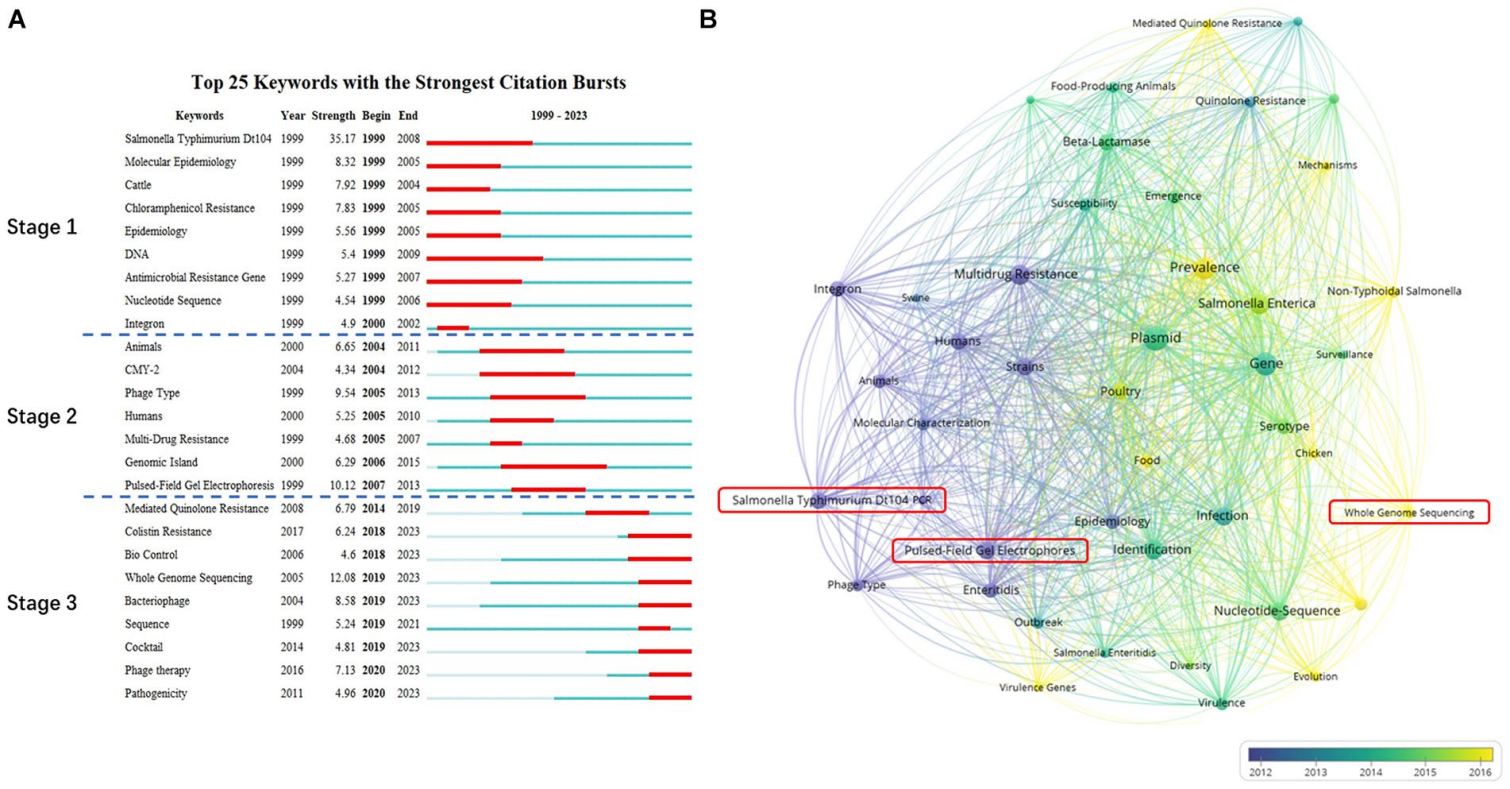
The evolution of keywords. **(A)** Top-25 keywords with the strongest citation bursts: “year” represents the first time the keywords appeared. “Begin” and “End” indicate the start and termination point of the period of heightened research activity on a specific keyword. “Strength” refers to the intensity of the burst during this period. **(B)** The co-occurrence and evolution of keywords: Node colors reflect the average publication year of each keyword, offering insight into the temporal distribution of keywords in the network. The size of circular nodes indicates the frequency of occurrence, with larger circles indicating a higher publication count. The thickness of the connecting lines represents the strength of the co-occurrence relationship, with thicker lines indicating a higher number of co-occurrences between two keywords.

Keyword burst detection refers to the significant increase in the frequency of keywords within a short period. The 25 keywords in the field with the strongest citation bursts are shown in Figure 3A. “*Salmonella* Typhimurium DT104” had the highest burst strength (35.17) from 1999 to 2008, followed by “Whole genome sequencing” (12.08) from 2019 to 2023, and “Pulsed-field gel electrophoresis” (PFGE) (10.12) from 2007 to 2013. This evolutionary trend of keywords was further visualized using VOSviewer (Figure 3B). “*Salmonella* Typhimurium DT104” and “Pulsed-field gel electrophoresis” are in purple with an average publication year before 2013 while “Whole genome sequencing” is in yellow with an average publication year after 2016.

## Discussion

To date, no bibliometric analysis has been conducted on HGT in *Salmonella* AMR. Therefore, we performed the first bibliometric analysis of this specialized area. The retrieved publications covered various aspects of HGT in *Salmonella* AMR, and these topics have been discussed in both livestock and human (Antunes et al., 2006; Mathew et al., 2009).

### Evolution of publications

According to de Price (1969), the Price’s index in physics and biology is approximately 60–70%, which is much higher than our result. The low Price’s index corresponds to a long cited half-life in this area, signifying a relatively gradual turnover rate. This illustrates the stability and maturity of the knowledge in this field over time. The citation/publication index shows the average quality of all publications, while the H-index reflects more about the number of high-quality publications (Hirsch, 2005). Several bibliometric analyses have focused on other topics in AMR, such as carbapenem resistance (Sweileh et al., 2016), AMR in food-producing animals (Sweileh, 2021), or AMR in the environment (Sweileh and Moh’d Mansour, 2020). In these fields, the citation/publication is approximately 22 (21.47, 22.4, and 22, respectively), which is lower than our result (29.23). However, the h-index for carbapenem resistance and food-producing animals’ AMR is 102 and 122, respectively, which are much higher than our results (87). This indicates that the overall quality of publications on HGT in *Salmonella* AMR is above the average level in the field of AMR, but the number of high-quality papers is still inferior to some of the other topics.

### The geographic distributions of publications

When we look into highly productive authors, institutions, and countries, the USA, China, and France are the three outstanding countries with different characteristics. The USA is widely recognized as a global leader and holds a prominent position in the scientific research area (These are the 10 best countries for life sciences research, 2020), which is the same in the field of HGT in *Salmonella* AMR. This was proven by the number of publications, citations, and highly productive authors and institutions in the USA. China is second to the USA in terms of the number of publications but with a low number of citations per publication. In recent years, China has significantly increased funding and policy support for scientific research (Marginson, 2022), which has led to an increase in publications from China (Figure 1). However, the emphasis on quantity rather than quality in some academic evaluation systems may lead to higher publication output without a sufficient emphasis on impact and scientific rigor (Wang et al., 2020). To address this, enhancing the quality-oriented evaluation system is important to improve the impact of Chinese research in this field. In contrast, France published only 78 papers in this field but with the highest citation/publication. The INRAE and Pasteur Institution are outstanding in terms of citation/publication. Both institutions are government research centers and have a profound history of bacterial research, which may explain the high quality of their scientific output.

### Evolution of research hotspots

When considering publication numbers, highly cited papers, and burstiness of keywords together, we identified three different stages from 1999 to 2024 and observed a clear evolution of research hotspots in the area of HGT in *Salmonella* AMR.

## Stage 1: understanding of MDR *Salmonella* Typhimurium DT104 (*S*. Typhimurium DT104) (1999–2003)

During this stage, the number of publications was less than 30 per year. According to the strength of the keyword citation burst, the research hotspot during this stage is to genetically explain the MDR mechanism of *S*. Typhimurium DT104 and how it transfers to other strains. MDR *S*. Typhimurium DT104 is known for its ability to cause infections in both humans and animals and has gained significant attention due to its emergence in the 1990s. These resistance genes can be horizontally transferred to other bacteria. Basic molecular methods and Sanger sequencing enabled the characterization of the integrative element *Salmonella* genomic island 1 (SGI1) and its MDR antibiotic gene cluster and demonstrated its potential for HGT.

During this stage, several highly cited publications provided fundamental experience for future research. Threlfall (2000) provided an overview of the international epidemic of MDR *S*. Typhimurium DT104, especially for genetic studies on the HGT of resistance genes. Briggs and Fratamico (1999) cloned and sequenced AMR genes that confer the ACSSuT-resistant phenotype. These genes were grouped within two district integrons and intervening plasmid-derived sequences. This sequence is potentially useful for detection MDR DT104. Doublet et al. (2005) discusses SGI1, which contains an AMR gene cluster found in various *Salmonella enterica* serovars. It demonstrated SGI1’s ability to be conjugally transferred and integrated into the chromosomes of recipient strains in a site-specific manner. This sheds light on the mechanisms underlying the spread of AMR genes, and highlights the potential of SGI1 to disseminate MDR among different bacterial populations. Boyd et al. (2001) first reported SGI1 in *S*. Typhimurium DT104 pandemic clone. Several open reading frames showed significant homology with plasmid-related genes, suggesting a plasmid origin for SGI1.

## Stage 2: application of PFGE and emergence of CMY-2 related β-lactamase resistance (2004–2017)

From 2004 to 2017, the number of publications per year was 30–70. During this period, PFGE was widely considered the “gold standard” technique for *Salmonella* genotyping (Neoh et al., 2019). After achieving a comprehensive understanding of *S*. Typhimurium DT104, the focus gradually switched to CMY-2 and related β-lactamase resistance. This may be related to the epidemic outbreak of the MDR *Salmonella* serovar Heidelberg from 2002 to 2005 in America and Canada (Zhao et al., 2008). This serovar substantially increased resistance to cephalosporins.

Resistance of *Salmonella* to expanded-spectrum cephalosporins has drawn the attention of the scientific community. *Salmonella* strains carrying *bla*_CMY-2_ were first isolated from human, animal, and food samples in the United States in 1996 (Zhao et al., 2001). In 2004, a comprehensive review of AMR in non-typhoid *Salmonella* is highly quoted by researchers (Su et al., 2004). This paper discusses how the gene *bla*_CMY-2_, which encodes extended-spectrum cephalosporinases in *Salmonella*, is transferred horizontally through conjugative plasmids, transposons, or integrons. The *bla*_CMY-2_ can be acquired through *in vivo* transfer from other pathogens in the intestines of patients. Additionally, resistance plasmids carrying *bla*_CMY-2_ can recombine with virulence plasmids to form hybrid plasmids that enhance *Salmonella* survival in drug environments and promote the spread of drug-resistant strains. Two studies reported the appearance of cephalosporin-resistant *Salmonella* expressing a plasmid-mediated CMY-2 AmpC β-Lactamase in 2000 and also received high citations (Dunne et al., 2000; Winokur et al., 2000). It is mainly carried by IncA/C plasmids, which have spread among different *Salmonella* serotypes and *E. coli* in all food-producing animals (Martin et al., 2012). Chen et al. (2004) also report plasmid-mediated transfer of genes encoding CMY-2 and TEM-1-like β-lactamases through conjugation studies. Further comparison showed that 19% of *Salmonella* isolates from retail meats purchased in the USA were resistant or exhibited intermediate susceptibility to ceftriaxone and harbored the *bla*_CMY-2_ gene. Conversely, all *Salmonella* isolates from China were susceptible to ceftriaxone (and other cephalosporins), and none harbored *bla*_CMY-2_. This may be linked to the earlier therapeutic use of cephalosporins in food animals in the USA than in China. In fact, after voluntary withdrawal of ceftiofur in 2005, resistance to ceftiofur declined by 89% in Quebec retail chicken meat (Dutil et al., 2010). This supports the hypothesis that fluctuations in ceftiofur resistance were most likely driven by common exposure (or reduction of exposure) to ceftiofur, rather than simply being secondary to the natural spread and disappearance of a ceftiofur-resistant clone unrelated to ceftiofur use.

## Stage 3: application of whole genome sequencing (WGS), emergence of quinolone resistance (*qnr*) and bio-control (2018–2023)

Since 2018, the annual number of publications has increased to over 70, and has reached more than 100 in 2021. “Whole genome sequencing,” “quinolone resistance,” “bio control” and “phage therapy” became key elements during this stage.

WGS has been increasingly recognized as a promising substitute for *Salmonella* typing (Ibrahim and Morin, 2018). In the post-genomic era, the affordability of the WGS technology has facilitated its widespread adoption in the research community. The analysis of WGS data has significantly enriched our understanding of the population structure, transmission dynamics, and host persistence. In a study with the highest number of citations, Kingsley et al. (2009) utilized WGS to analyze the genetic makeup of the MDR ST313 NTS isolate, D23580, as well as other epidemic ST313 isolates from Malawi and Kenya. Researchers have identified distinct prophage repertoires, composite genetic elements encoding MDR genes, and evidence of genome degradation including pseudogene formation and chromosomal deletions. This suggested that the virulence plasmid may act as a platform for capturing AMR genes, facilitating the exchange of genes collected from other bacteria in the environment. This approach allowed for a comprehensive analysis of the genetic basis of AMR and virulence potential in the studied *Salmonella* Typhimurium strains. Therefore, WGS plays a crucial role in providing detailed insights into the genomic characteristics of the epidemic ST313 NTS isolate and its implications in AMR and pathogenicity.

There has been a growing interest in *Salmonella* plasmid epidemiology due to the emergence of plasmid-mediated *qnr* genes (Gay et al., 2006), the sporadic spread of plasmid-borne ESBL genes (Dor et al., 2020), and the development of plasmid typing methods (Carattoli et al., 2005). In 2006, Gay et al. (2006) first report of plasmid-mediated *qnr* in *Salmonella* isolates from the United States and arouse high citations. This study emphasizes the potential for rapid spread of plasmid-mediated fluoroquinolone resistance and simultaneous resistance to multiple classes of antimicrobial agents. In addition, the presence of multiple *qnr* variants in several *Salmonella* serotypes from widely separated states suggests a broad host and geographic distribution, raising concerns regarding the insidious spread of resistance and the potential for therapy-threatening co-transmission of extended-spectrum β-lactamases.

The serious MDR status of *Salmonella* populations has made “the bio control” an important research area in recent years to prevent *Salmonella* colonization and transmission (Guenther et al., 2012). For example, phage therapy is considered a promising approach for combating *Salmonella* infections (LeLièvre et al., 2019). Bacteriophages specifically target and infect bacteria, hijack the bacterial machinery to replicate, and ultimately lead to cell lysis. However, bacteriophages can act as vehicles for HGT by carrying bacterial DNA during the infection cycle (Borodovich et al., 2022). Thus, HGT may contribute to the spread of AMR in bacterial populations (Colavecchio et al., 2017). The potential of HGT in bacteriophage therapy is an area of active research that requires careful consideration to mitigate any adverse effects, such as the dissemination of AMR genes.

## Conclusion

In summary, investigation of HGT in the context of *Salmonella* AMR represents a dynamic and internationally collaborative research domain. The enduring stability and maturity observed in this field underscores its status as a firmly established and influential area of study. Novel methods and technologies such as high-throughput long-read sequencing and advanced bioinformatics tools present exciting opportunities to deepen our understanding of HGT in *Salmonella* AMR. For instance, the use of PacBio long-read sequencing in characterizing 134 *Salmonella* isolates from raw meats and food animals has elucidated the genomic structure and location of resistance genes, significantly contributing to our knowledge of HGT in *Salmonella* (Li et al., 2021). Also, the application of an ISO-certified genomics workflow for identifying and validating antimicrobial resistance in *Salmonella* spp. demonstrates the effectiveness of advanced bioinformatics tools in this field (Sherry et al., 2023). In addition, bio-control methods can act as a novel and effective strategy to combat the rising threat of MDR *Salmonella* strains. For example, the use of bacteriophages in treating antibiotic-resistant *Salmonella* infections has shown promising results, with engineered phage cocktails achieving clinical improvements in a significant number of cases (Khan and Rahman, 2022). Similarly, probiotics such as *Bacillus subtilis* has been found to inhibit the formation of *Salmonella* biofilms, offering an alternative to traditional antibiotic therapies (Jeon et al., 2017).

## Data Availability

The original contributions presented in the study are included in the article/supplementary material, further inquiries can be directed to the corresponding author.
